# Supplementary material for: Deep learning innovations in South Korean maritime navigation: Enhancing vessel trajectories prediction with AIS data
Source: PLoS One. 2024 Oct 24;19(10):e0310385. doi: 10.1371/journal.pone.0310385 (PMC11500964; doi:10.1371/journal.pone.0310385)
Supplement: Attachment — Submitted filename: Response to Reviewers.docx [file pone.0310385.s002.docx]

**Reviewer #2 Comments (Round-2)**

**Reviewer #2:**

**Concern #1: The authors have successfully addressed the majority of the previous comments. However, I have noticed that some abbreviations are repeated unnecessarily throughout the manuscript. It would be beneficial to define each abbreviation only once when it first appears in the text and use the abbreviation consistently thereafter. Additionally, the manuscript's formatting is inconsistent, particularly in the justification of the text. Ensuring that the text is uniformly justified will enhance the readability and overall presentation of the paper. These are minor issues that should be corrected before final acceptance.**

**Author Response:**

**Thank you for your insightful feedback regarding the formatting and abbreviation usage in our manuscript.** We have carefully reviewed the manuscript and made the necessary adjustments to ensure consistency in abbreviation usage, defining each abbreviation only once when it first appears in the text. Additionally, we have addressed the formatting issues by uniformly justifying the text throughout the document, enhancing the readability and overall presentation of our paper. We believe these revisions improve the clarity and quality of the manuscript.

**ACADEMIC EDITOR:**

**Please define each abbreviation only once when it first appears in the text and use the abbreviation consistently thereafter. Also, please check the reviewer 2 comments regarding the formatting.**

**Author Response:**

We appreciate the Academic Editor's feedback and have thoroughly reviewed the manuscript to ensure that all abbreviations are defined at their first mention and are used consistently throughout the text. We understand the importance of maintaining consistency in abbreviation usage for clarity and have implemented the necessary changes.

In response to Reviewer 2's comments regarding the formatting, we have also ensured that the text is uniformly justified throughout the manuscript. We believe these revisions will significantly enhance the readability and presentation of our paper.
